# Detection of aberrant locomotor activity in a mouse model of lung cancer via home cage monitoring

**DOI:** 10.3389/fonc.2024.1504938

**Published:** 2024-12-23

**Authors:** Michele Tomanelli, Federica Guffanti, Giulia Vargiu, Edoardo Micotti, Mara Rigamonti, Francesca Tumiatti, Elisa Caiola, Mirko Marabese, Massimo Broggini

**Affiliations:** ^1^ Department of Experimental Oncology, Istituto di Ricerche Farmacologiche Mario Negri IRCCS, Milan, Italy; ^2^ Department of Neuroscience, Istituto di Ricerche Farmacologiche Mario Negri IRCCS, Milan, Italy; ^3^ Tecniplast SpA, Buguggiate, Italy

**Keywords:** KRAS/LKB1, NSCLC, locomotion, home cage monitoring, biomarker, transgenic animal models, MRI, translational models

## Abstract

**Introduction:**

Lung cancer is the first cause of cancer death in the world, due to a delayed diagnosis and the absence of efficacy therapies. KRAS mutation occurs in 25% of all lung cancers and the concomitant mutations in LKB1 determine aggressive subtypes of these tumors. The improvement of therapeutical options for KRASG12C mutations has increased the possibility of treating these tumors, but resistance to these therapies has emerged. Preclinical animal models permit the study of tumors and the development of new therapies. The DVC system was used to measure circadian activity changes indicative of lung cancer progression in KRAS and KRAS-LKB1 transgenic mouse models.

**Material and methods:**

KRAS and KRAS-LKB1 conditional transgenic animal models were bred and genotyped. The tumors were inducted using adeno-CRE-recombinase system. The mice were housed in a Digital Ventilated Cage (DVC^®^) rack measuring the locomotor activity continuously for 24/7. The progression of the tumors was monitored with MRI. The DVC system evaluated a reduction in animal locomotion during the tumor progression.

**Results:**

KRAS and KRAS-LKB1 mutations were induced, and the tumor formation and progression were monitored over time. As expected, the onset of the tumors in the two different breeds occurred at different times. DVC system registered the locomotion activity of the mice during the light and dark phases, reporting a strong reduction, mainly, in the dark phase. In KRAS-LKB1 models, the locomotion reduction appeared more pronounced than in KRAS models.

**Discussions:**

Transgenic animal models represent a fundamental tool to study the biology of cancers and the development of new therapies. The tumors induced in these models harbor the same genotypical and phenotypical characteristics as their human counterparts. DVC methods permit a home cage monitoring system useful for tracking animal behavior continuously 24 hours a day, 7 days a week. DVC system could determine disease progression by monitoring a single animal activity in a cage and also using group-housed animals. For these reasons, the DVC system could play a crucial role in identifying diseases at early stages and in testing new therapeutic approaches with a higher likelihood of efficacy.

## 1 Introduction

Lung cancer is the first cause of cancer death in men and the second in women (1–3). In most cases the disease manifests in an advanced stage, whereas only in 20% of patients the pathology is diagnosed at an early stage (4, 5). The clinical picture is further complicated by the lack of fully effective pharmacological therapies. The 5-year overall survival for these patients decreases depending on the stage at which the tumor is diagnosed (6, 7). Lung cancer is considered an environmental disease and tobacco is the principal risk factor for this pathology (8, 9). However, lung cancer can also occur in non-smokers due to various causes, and these tumors exhibit different biochemical characteristics compared to those found in smokers (10–12). The advancement of molecular biology techniques has improved the knowledge about the biology of tumors and the main mutations that characterize these malignancies. KRAS mutations represent 25% of all lung cancers (13–16). KRAS-LKB1 co-mutated tumors are a frequent and very aggressive subtype of these tumors, and in particular in non-small-cell-lung-cancer (NSCLC) (17–25). In particular, about 50% of KRAS-mutated lung cancer exhibit also deletions in LKB1. LKB1 is a kinase involved in metabolic control, redox homeostasis, and cell polarity (19, 22, 26, 27). LKB1 activates AMPK, a cellular nutrient sensor capable of detecting the ATP/ADP ratio to determine the cell’s energetic status (28, 29). This pathway is closely linked to the PI3-AKT-mTOR pathway, with which it has an opposing functional relationship. While the LKB1-AMPK pathway metabolically favors the activation of catabolic reactions, the latter promotes protein synthesis and anabolism in general. When the cell is in an energy deficit (low ATP/ADP ratio), the LKB1 pathway is activated, shutting down AKT-mTOR signaling to promote catabolic cellular reactions, aiming to restore proper cellular energetic status. In tumors, LKB1 is deleted, leading to the constitutive activation of the PI3K-AKT-mTOR pathway, resulting in a growth advantage for the tumor. KRAS modulates the activation of PI3K-AKT-mTOR pathway, therefore, the concomitant mutation between KRAS and LKB1 determine ad advantage for the tumor growth. The mutational spectrum of KRAS is particularly diverse, but the most frequent mutation in lung cancer is G12C (30–32). For many years, despite numerous attempts, KRAS mutations have posed a significant obstacle to the treatment of these patients, in fact, the only therapeutical approach was conventional chemotherapy and the prognosis was poor (33, 34). In recent years, significant advancements in treating lung cancer subtypes with KRAS G12C mutations have led to an increase in patient survival (35–40). However, as for the other targeted therapy, resistance to these therapies has already been observed at the clinical level (41–45). The absence of a fully effective therapies for treating these tumors necessitates further research to explore new therapeutic approaches. Preclinical animal models are a crucial tool for enhancing our understanding of tumors and for exploring new therapeutic strategies (46–50). These models represent an intermediate step in the experimentation process between cell cultures and patients. In particular, transgenic animal models allow for the study of specific mutations that define different tumors (51, 52). Transgenic animal models develop tumors that maintain the same characteristics as patient tumors, allowing the results to be translated into clinical practice (53–55). Furthermore, in these models, is possible to reproduce the different phenotypes of the disease. In this work, we inducted the onset of the tumors in KRAS and KRAS-LKB1 transgenic mice following the pathogenesis over time (53, 56–58). Mice with the co-mutation in KRAS/LKB1 develop more aggressive tumors, as the deletion of LKB1 increases the potential for metastasis (53, 59–62). The goal of these experiments was to observe the motor activity of the mice using the DVC ventilated cage system. DVC system allows the evaluation of these parameters without disturbing the animals. The development of tumors in the lung lobules of the animals over time was monitored using magnetic resonance imaging (MRI) (17). This tool (DVC system) could represent a significant advancement in understanding tumor biology, as well as improving the ethical standards of experiments.

## 2 Material and methods

### 2.1 Maintenance of conditional transgenic mice colonies

Conditional transgenic animal models were acquired from the Jackson Laboratory in two different strains: c57-B6.129S4-KRAS^<tm4Tyj>/J^ and c57-Stk11^tm1.1Sjm/j^. The first strain was modified in KRAS gene for expressing the mutation G12D. The second strain was modified in LKB1 gene to delete the protein. The animals were mated (2 female and 1 male) to expand the colonies of every strain. After 3 weeks postpartum female and male mice were weaned and separated in different cages. The identification of the mice was permitted by inserting a numerical tag into the ear and simultaneously taking a small tissue sample (from the ear); the samples were necessary to genotype the animals. KRAS and LKB1 mutated mice were crossed to generate colonies that harbor both mutations. The genotype of interest was KRAS het/LKB1 ko. However, we also carried out breeding with transgenic mice modified only in the KRAS gene, in order to subsequently develop tumors with the single KRAS mutation (het). Since these models are conditional mice, at this stage the mutations of interest were not yet present; they were only expressed after the administration of Cre-recombinase (SignaGen Laboratories, Rockville, MD, USA). In the case of LKB1, it involves a gene deletion, thus a knock-out, whereas in the case of KRAS, it involves the acquisition of a point mutation, thus a knock-in.

### 2.2 Genotyping PCR

Tissue samples (small ear pieces) were collected from the mice during weaning. DNA was extracted from the samples using Maxwell ^®^ 16 Tissue DNA Purification (Promega). The DNA extracted was quantified using NanoDrop 2000 (Thermo-Fisher). The 260/280 ratio of the samples ranged between 1.8 and 2.0. PCR was performed using a hot-start DNA polymerase mix (Promega) containing oligonucleotides, reaction buffer with dye, and other reagents important for the function of DNA polymerase. Primer sequences specified for KRAS-LKB1-modified genes were provided directly from Charles River. In the same way, the PCR program was supplied by the company. KRAS-PCR showed a single amplified band (250 bp) when the genotype was wild-type, instead, resulted in two bands (250 bp and 100 bp) when the genotype was heterozygous for G12D mutation. LKB1-PCR highlighted three possible variants: a single band to 348 bp when the genotype was wild-type, two bands to 348 bp and 450 bp when the genotype was heterozygous and, a single band to 450 bp when the genotype was homozygous for the mutation. KRAS transgenic mice were a knock-in model, whereas LKB1 transgenic mice were a knock-out model.

### 2.3 Lung tumors induction with CRE-recombinase system

After 4 weeks from birth the mice carrying the modified alleles (KRAS het/LKB1 ko) were anesthetized with ketamine (75 mg/kg) and medetomidine (1 mg/kg). The animals were inoculated with adenoviral CRE-recombinase. The viral particles used for the induction were quantified in 5*10^6^ for every animal. The administration of viral particles was performed in intranasal way. The mice were anesthetized with medetomidine and ketamine. Subsequently, under sterile conditions, the virus was delivered directly into the animals’ nostrils. After inoculation, the mice were awakened using an antidote. The animals were then kept in ventilated cages for a few days to allow the viral load to dissipate. For some animals, we attempted to administer the virus directly in the trachea, but due to the difficulties related to animal handling, we decided to continue exclusively with intranasal administration.

### 2.4 Magnetic resonance to monitor tumor development

During the MRI scans (duration 20-30 min/mouse), animals were kept under controlled anesthesia conditions (100% O2, 2% isoflurane). The animals’ temperature was kept at 36.5 +/-0.5°C using a warmed cradle. Mouse lungs were scanned with a T2w sequence using a respiration triggered spin-echo sequence (12 coronal slices of 1 mm thickness, TR/TE = 1000/25 ms, 117 µm2 in-plane resolution, FOV 3x2 cm^2^, matrix size = 256 x 172).

### 2.5 Quantification of the lung volume occupied by the tumor

Images have been analyzed using two free software: Fiji (63) and ITK-SNAP (64). The whole lung has been manually segmented and extracted from the whole chest image stack. The signal of the dark healthy tissue has been set as background. After that a threshold of 3 standard deviations of the background was applied in order to highlight the tumoral tissue with respect to the healthy one. The mask determined in this way covered the tumoral tissue inside the lungs. At the end of the automatic segmentation, an expert researcher has checked the goodness of the segmentation. To consider the different lung sizes between subjects, the volume of the tumor has been expressed as a percentage of the whole lung volume.

### 2.6 DVC methods

30 animals were housed in a Digital Ventilated Cage (DVC^®^) rack, a home cage monitoring system capable of continuously tracking animal activity 24/7. This system builds up upon a standard IVC rack by placing an electronic capacitance sensing board beneath each cage to detect the movement of the animals in the area surrounding each of the 12 electrodes that compose the board (65). We used 8 cages, with each cage containing between 3 and 5 animals. Five of these cages contained KRAS/LKB1 mice, while three cages contained KRAS mutant mice. We used the Animal Locomotion Index Smoothed (DVC^®^ Analytics, Tecniplast S.p.A., Buguggiate, Italy) to measure the activity of the mice. This metric is based on the activation density metric defined in (65) which calculates the number of electrodes activations caused by mouse activity, and normalizes it by dividing by the time interval and the number of electrodes (yielding values ranging from 0 to 1). We averaged activity by day and/or week, by distinguishing the light and dark phases of the day. Since the cage numerosity was different between cages and changed over time (because of the separation of some animals due to fight, especially among males), we normalized the animal activity by dividing by the number of the animals in each cage at any given time. Finally, we performed a linear regression analysis on the daily activity data to quantify trends in activity over time.

### 2.7 Statistical analyses

We used Python to process and visualize DVC data. We used scikit-learn package to fit a linear regression model on daily activity data over time (light and dark separately). We used R to run the corresponding statistics, with significance level α = 0.05. We used lme4 and lmerTest R software packages to model weekly activity data as Linear Mixed Model and test for fixed effects of genotype and time, and a random effect of time (**Supplementary Tables S1**, **S2**). We tested the normality assumption of the model residuals and random effects with Shapiro-Wilk tests (p>0.05), and the homoscedasticity of the model residuals with a between-subjects one-way ANOVA (p>0.05).

## 3 Results

### 3.1 Generation and maintenance of murine colonies harboring KRAS and KRAS/LKB1 mutation

KRAS and LKB1 mutations frequently characterize NSCLC (17, 22). As reported in the literature, transgenic animal models with LKB1 mutations do not develop tumors. The presence of co-mutation in KRAS and LKB1 instead determined the onset of tumors in the models. Furthermore, these tumors are very aggressive and prone to metastasis. Transgenic animal models with mutations only in the KRAS gene can also develop tumors. Therefore, we decided to study these mutations with transgenic animal models. The animals used were inducible because the modified genes were floxed and only the administration of cre-recombinase can activate the phenotype (66). Therefore, at this stage the animals bred were wild-type. In the LKB1 model, alterations are present in both alleles of the gene, whereas in the KRAS model, the mutation was present in only one allele, because the mice with mutations in both alleles do not survive to birth. The two strains were expanded to determine enough mice to start the crosses. The crosses were maintained for three generations to stabilize the genotype. Before beginning the crosses between the two different mouse strains, the theoretical allele frequencies were determined. After starting the crosses the real allele frequencies were determined to check whether the expected frequencies were confirmed. **Table 1** reported the allele frequencies.

**Table 1:** The table reported the expected genotypic frequencies on our transgenic models, alongside the real frequencies observed in a total of approximately 200 animals.

Genotype	Expected frequencies	Observed frequencies
KRAS WT-LKB1 WT	6.25%	11.5%
KRAS HET-LKB1 WT	12.5%	14.54%
KRAS WT-LKB1 HET	12.5%	12.12%
KRAS HET-LKB1 HET	25%	24.84%
KRAS WT-LKB1 KO	6.25%	16.9%
KRAS HET-LKB1 KO	12.5%	20%

The first column reports the genotype of the animals. The second column reports the expected frequencies crossing these genotypes. The third column shows the frequencies obtained after starting the breeding crosses. The observed frequencies largely match the expected ones for most genotypes.

### 3.2 Induction of the mutations and tumor timing

The mutations in the animals were inducted with Cre-recombinase administrating 5*10^6^ viral particles. This number of viral particles was chosen because it results in a level of lesions that is not too extensive and therefore difficult to quantify. Both the strains (KRAS mutated and KRAS-LKB1 mutated) were treated with this number of viral particles. The mice were treated with the virus after 4-6 weeks from birth because older mice were less prone to develop lesions. The timing of the onset of the tumors was determined through pilot experiments using MRI protocol. The mice were weekly monitored identifying the first lesions after 3 weeks from the virus administration in KRAS-LKB1 mice. The mice with only KRAS mutation displayed the first lesions a few weeks later (10-12 weeks) after injection.

### 3.3 Timing of tumor onset

**Figure 1:**
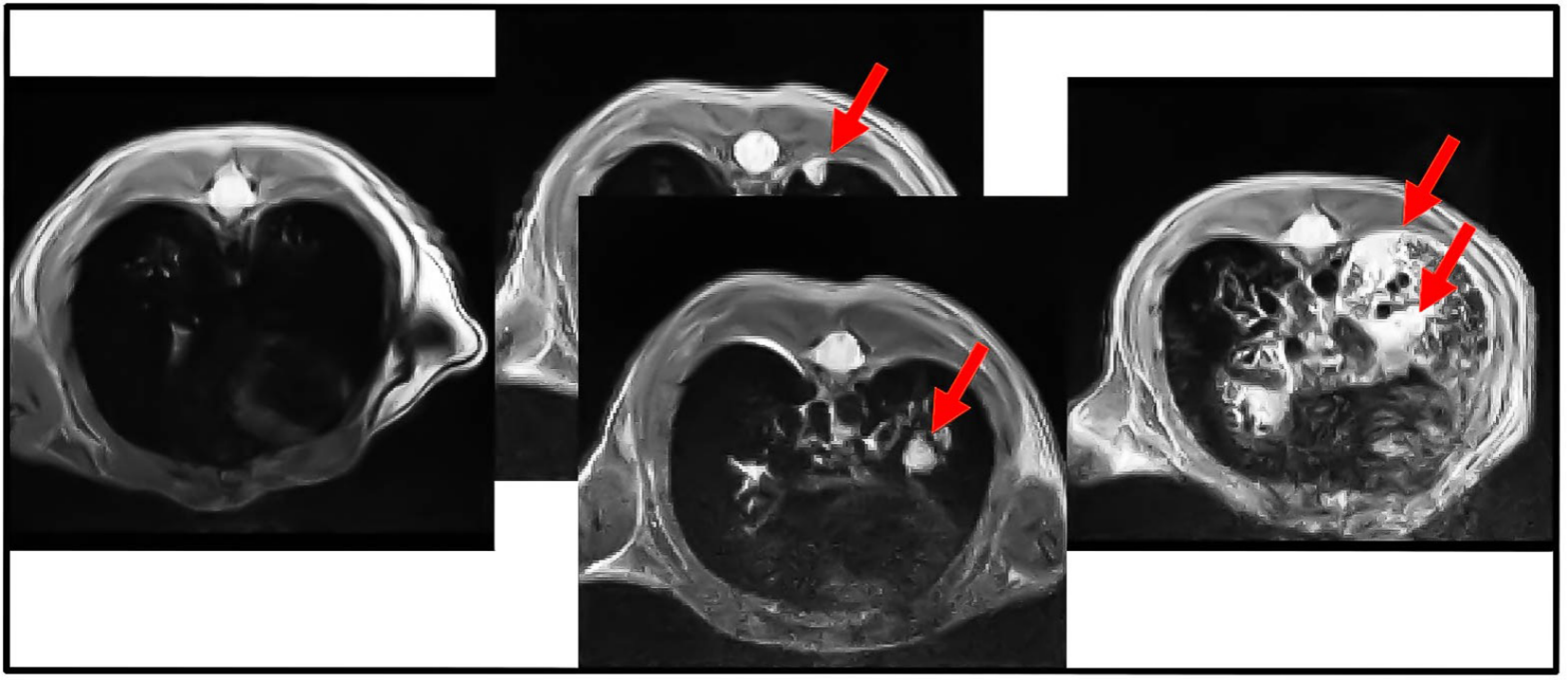
Cross-section of MRI analysis of the lungs from KRAS/LKB1 mutated mice. The arrows show the presence of nodules. Images were acquired weekly for 4 weeks, showing the progression of lesions over time. The arrows indicate the presence of nodules in the lungs. This scan was derived only from KRAS/LKB1 mice. The first scan was referred to T0. The scan in the middle was referred to T1. The last scan was referred to T2.

The onset of the tumors was observed using magnetic resonance (MRI). The injection of the viral particles should be carried out after 4-6 weeks from the birth. This timing was important because older animals could be less susceptible to the onset of the tumor with this method. Furthermore, the presence of elevated adipose tissue may increase anesthetic-induced toxicity (mainly in males). The figure below (**Figure 1**) shows some images captured by MRI and the presence of the tumor lesions increased after each week. The quantification of the lesions has been performed with two software (ITK-SNAP and Fiji), as reported in the methods. These parameters were measured to plan the main experiment.

### 3.4 Digital ventilated cages system analyses evaluated the locomotion activity of the animals with tumor lesions

Normally, body weight is used as an endpoint to evaluate the health status of animals with tumor lesions in the lungs (17, 67, 68). When body weight decreases, it often indicates the presence of numerous lung nodules, as the weight loss is proportional to the number of lesions. However, this requires handling the animals. DVC analyses provide an alternative by using a different endpoint to monitor disease progression unintrusively. The animals were placed in a ventilated cage system that measures their activity levels. The mice, in which the disease was induced by the injection of an adenoviral vector, were placed in the cage for monitoring. This experiment involved mice with KRAS/LKB1 mutations and those with KRAS mutations alone. The KRAS/LKB1-mutated model developed the disease more slowly than the KRAS-mutated mice. Specifically, the first lesions appeared 10-12 weeks after injection, as determined by MRI analysis. The monitoring of KRAS/LKB1-mutated mice was later placed in the cage due to the delayed onset of lesions.

### 3.5 Magnetic resonance analyses showed the presence of tumor nodules in the lungs

**Figure 2:**
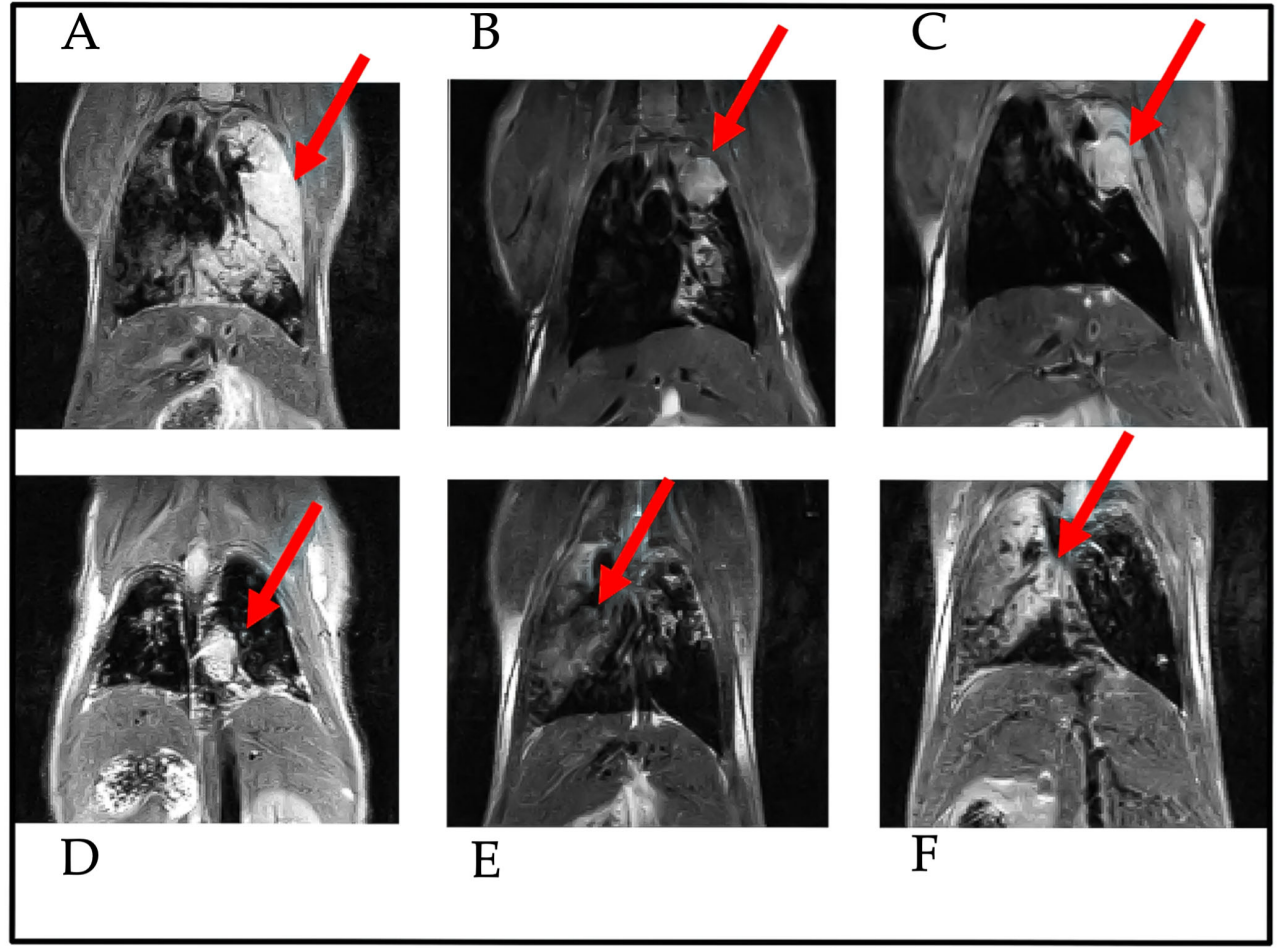
Frontal-section of MRI analysis of the lungs from KRAS/LKB1-mutant mice and KRAS-mutant mice. Images were captured 55 days after the initiation of DVC analysis. The arrows indicate the presence of nodules in the lungs. These scans were performed for both genotypes. The scans **A–D** were derived from KRAS/LKB1 mice. The last two scans **(E, F)** were derived from KRAS-mutated mice.

7 animals were also observed through MRI during the experiment to verify tumor onset. The nodules in the animals lung were quantified using the procedure described in the methods. The **Table 2** shows the percentage of lung tissue occupied by the lesions. **Figure 1** illustrates the cross-section of the animal lungs, clearly displaying the nodules occupying the lung volume. **Figure 2** depicts the frontal sections of the lung from KRAS/LKB1 mice and KRAS mice. Tumor lesions are clearly visible in all the animals observed.

**Table 2:** The table shows the percentage of the lesions present in the lungs of the animal relative to the lung volume.

Mice	Lung volume (mm^3^)	Tumor volume (mm^3^)	% tumor
157	1421	704.5	49.50%
159	1509	717	47.50%
423	1172	209.3	17.80%
426	1078	197.1	18.20%
435	1396	315	22.56%
460	1269	317	24.98%
474	1861	1068	57.38%

The first column reports the identification number of the animals. The second column shows the total lung volume of the animals (mm^3^), segmented using the programs applied for quantification. The third column shows the volume of tumors (mm^3^) that occupied the lung of the animals. The fourth column shows the percentage of tumor volume relative to the total lung volume.

### 3.6 Activity from home-cage monitoring

**Figure 3:**
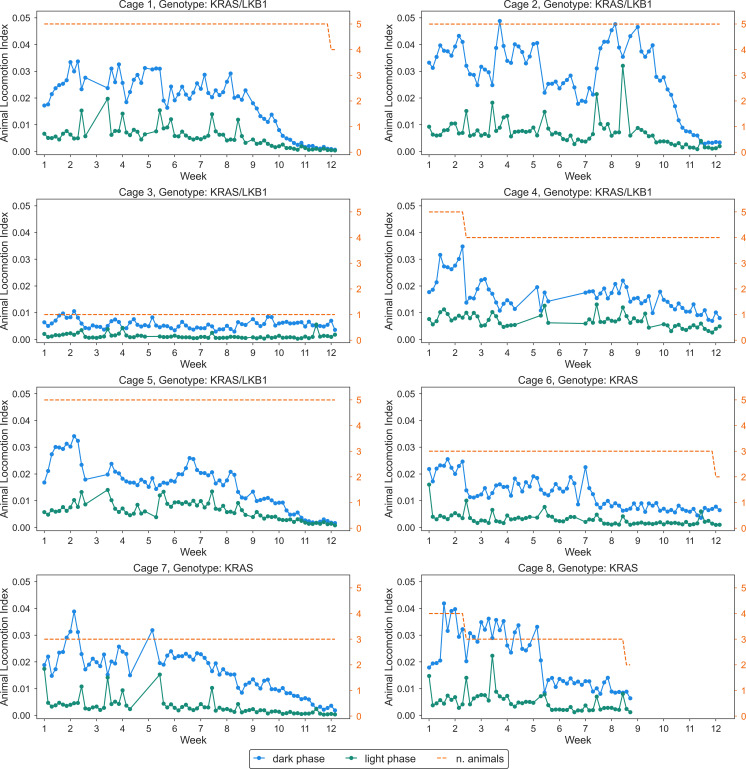
Daily activity of all the cages (KRAS/LKB1 and KRAS) in light and dark conditions. Each panel depicts daily average activity across 81 days, in cages of KRAS/LKB1 and KRAS mice. KRAS/LKB1 mutated mice started the measurement after about 28 days from the birth, while KRAS mice started the measurement after about 60 days from the birth. The blue lines show the activity during the dark phase, while the green lines represent activity during the light phase. The dashed orange line and axis represents the number of animals inside the cage, which changed over time.

**Figure 4:**
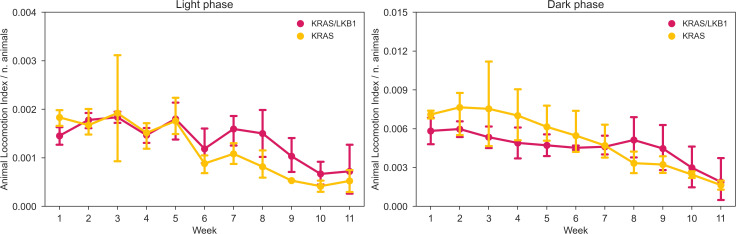
Average weekly activity normalized per cage density. Average weekly normalized activity (± SD) over light (left panel) and dark (right panel) phases across 11 weeks of observation and between two genotypes. N of cages per group: KRAS/LKB1 = 5, KRAS = 3.

**Figure 5:**
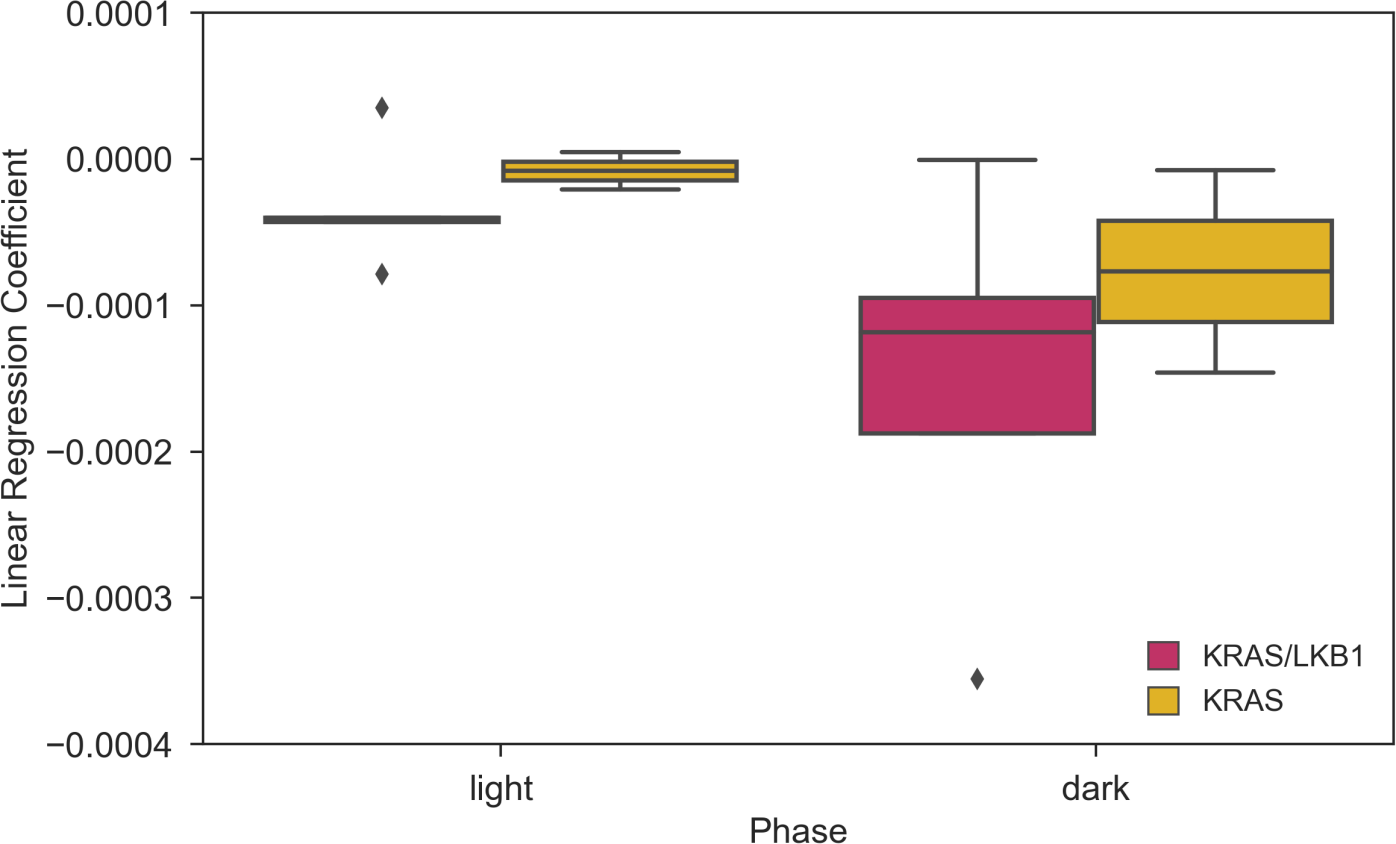
Coefficients of the Linear Regression analysis of daily activity over time during weeks 8-11. Slope of the linear regression computed for the day and night time daily activity points in weeks 8-11. N of cages per group: KRAS/LKB1 = 5, KRAS = 2.

The activity of the animals was monitored by the DVC^®^ system 24/7 for 81 days. **Figure 3** shows the average daily activity of 5 cages of KRAS/LKB1 mice and 3 cages of KRAS-mutated mice, during the light and dark phases over time. As expected, all the mice displayed a higher activity during the dark phase, which is consistent with their nocturnal behavior (69). The graphs show a reduction in movement starting at around 50-60 days, especially during the dark phase. **Figure 4** displays the average weekly activity of the mice during the light and dark phases. The activity has been normalized by the number of mice, due to differences in cage numerosity between units and over time. We observed a decline in activity across individual genotypes in both light conditions (lmerTest, *p_week_* <0.001). During dark phase, KRAS/LKB1 mice generally showed lower activity compared to KRAS-mutated mice (lmerTest, *p_genotype_* <0.05), but a different evolution over time (lmerTest, *p_genotype×week_* <0.05). The details of the Linear Mixed Models are reported in the Supplementary Materials (**Supplementary Tables S1**, **S2**). We quantified the decrease in activity over the last observation month by performing a Linear Regression on daily activity data starting from week 8 (considering only the cages present during that period). **Figure 5** shows the slope coefficients in light and dark phases. While the decrease of activity during lights-on is absent with a slope close to 0 in both genotypes, during the dark period KRAS/LKB1 exhibit a lower coefficient than KRAS mice. This suggests a faster decrease in activity, although statistical testing was not possible due to the limited sample size in the final observation weeks.

## 4 Discussion

In this study, transgenic animal models of lung cancer harboring KRAS/LKB1 and KRAS mutations were used. Despite advancements in understanding the molecular biology of these tumors and the development of new therapeutic options, these tumor subtypes remain lethal. In several circumstances, the aggressiveness of the tumors harboring mutations in KRAS/LKB1 precludes the possibility to perform a proper treatment for the patients. It is, therefore, mandatory to have preclinical models able to recapitulate the human tumors. These models can help in defining new treatments for these unfavored tumors (58, 70, 71). Examples of patients derived xenografts, i.e. tumors obtained at surgical level from patients and implanted in immunodeficient animal are an important tool. However, one of the drawbacks of these important models is the lack of a full immune system that does not allow the testing of immune checkpoint inhibitors that represent an important armamentarium for NSCLC patients. In this context, transgenic animal models that are obtained in fully immunological competent mice, represent important tools for enhancing cancer research. Specifically, LKB1/KRAS transgenic animal models serve as translatable models for human lung cancer due to the similar molecular and histological characteristics of the tumors. However, these models can generate different histological subtypes, reflecting the typical heterogeneity of cancer. Additionally, KRAS-mutated transgenic animal models were employed in this study. This model can produce tumors, although they are less aggressive compared to the KRAS/LKB1 model. As expected, tumor onset occurred at different times in the two models. In our study, we also attempted to use a method that can quantify lesions within the animals’ lungs. This quantification method could be useful in the future for studying tumor progression. MRI analysis is a fundamental technique for examining the lungs of animals to assess the presence of lesions; however, this technique requires animal manipulation. The DVC methods represent a home cage monitoring system designed for continuous tracking of animal behavior 24 hours a day, 7 days a week (72–74). Specifically, the Animal Locomotion Index Smoothed was used to measure the activity of the mice. A recent study conducted on lung tumor models demonstrated that DVC methods could determine disease progression by monitoring the activity of individual animals in a cage (75). The analyses conducted with the DVC demonstrate a significant decrease in the locomotor activity of the animals during disease progression. The ability to analyze the animals with MRI also confirmed the presence of tumor lesions within the lungs of the animals. However, the absence of a healthy control group represents a limitation in the interpretation of our data, and a comparison in the evaluation of locomotor activity between animals without lesions and animals with tumors would certainly have strengthened our findings. In KRAS/LKB1 models, the locomotion reduction appeared more pronounced than in KRAS mice, possibly reflecting the aggressive nature of these tumor subtypes. However, this remains a hypothesis, as we lack statistical evidence to confirm that KRAS7LKB1 mice exhibit a greater reduction in locomotion compared to KRAS mice due to the more aggressive nature of the former tumors. This study represents a significant advancement, as the DVC system can detect variations in animal activity related to disease progression while using group-housed animals (76, 77). Importantly, a key feature of the DVC system is its ability to monitor disease progression without disturbing the animals. The possibility of using the DVC on other tumor models is a goal for the future. In particular, considering the high potential of conditional transgenic mouse models, which allow for the replication of all mutations that characterize tumors, the same methodology could be applied to conduct similar evaluations in many other types of cancers, not just lung cancer. For example, a study conducted on breast cancer tests the possibility of using the DVC system to evaluate new analgesics aimed at reducing tumor-induced pain in animals (78). These findings pave the way for developing new therapeutic strategies, whose effectiveness could potentially be detected in early stages, increasing the likelihood of achieving the desired outcomes.

## Data Availability

The raw data supporting the conclusions of this article will be made available by the authors, without undue reservation.
