# Supplementary material for: Detection of aberrant locomotor activity in a mouse model of lung cancer via home cage monitoring
Source: Front Oncol. 2024 Dec 23;14:1504938. doi: 10.3389/fonc.2024.1504938 (PMC11701213; doi:10.3389/fonc.2024.1504938)
Supplement: Supplementary file 1 [file DataSheet1.pdf]

Detection of aberrant locomotor activity in a mouse model of lung cancer via home cage monitoring

Supplementary Information

Formula: activity ~ week + genotype + genotype:week + ((1 + week) | cage)					
Fixed effects:					
	Estimate	Std. Error	df	t value	Pr(>|t|)
(Intercept)	2.140e-03	1.628e-04	7.921e+01	13.146	< 2e-16 ***
week	-1.587e-04	2.726e-05	3.500e+01	-5.823	1.32e-06 ***
genotypeKRAS/LKB1	-1.857e-04	2.036e-04	7.808e+01	-0.912	0.3644
week:genotypeKRAS/LKB1	6.181e-05	3.330e-05	3.484e+01	1.856	0.0719

Table S1 – Statistical results of Linear Mixed Model for lights-on weekly activity.

Formula: activity ~ week + genotype + genotype:week + ((1 + week) | cage)					
Fixed effects:					
	Estimate	Std. Error	df	t value	Pr(>|t|)
(Intercept)	8.871e-03	6.889e-04	8.187e+00	12.877	1.02e-06 ***
week	-6.201e-04	8.942e-05	8.104e+00	-6.935	0.000113 ***
genotypeKRAS/LKB1	-2.450e-03	8.657e-04	8.000e+00	-2.830	0.022158 *
week:genotypeKRAS/LKB1	3.116e-04	1.095e-04	7.533e+00	2.845	0.023031 *

Table S2 - Statistical results of Linear Mixed Model for lights-off weekly activity.
